# Contrasting effects of climate and population density over time and life stages in a long‐lived seabird

**DOI:** 10.1111/1365-2435.12831

**Published:** 2017-04-03

**Authors:** Rémi Fay, Christophe Barbraud, Karine Delord, Henri Weimerskirch

**Affiliations:** ^1^ Centre d'Etudes Biologiques de Chizé UMR 7372 CNRS/Univ La Rochelle Villiers‐en‐Bois 79360 France

**Keywords:** *Diomedea exulans*, early‐life vital rate, juvenile survival, long‐term effect, recruitment age

## Abstract

1. Although population responses to environmental variability have been extensively studied for many organisms, few studies have considered early‐life stages owing to the inherent difficulties in tracking the fate of young individuals. However, young individuals are expected to be more sensitive to environmental stochasticity owing to their inexperience and lower competitive abilities. Thus, they are keys to understand demographic responses of an age‐structured population to environmental variability.
2. In this study, we used capture–recapture modelling, based on a 49 year‐long individual‐based longitudinal monitoring dataset, to investigate climatic and population density effects on immature demographic parameters in a long‐lived seabird, the wandering albatross.
3. We provide evidence that climate and population size affected both survival and recruitment age of young individuals although in different ways according to the trait. We found that early‐life survival was mainly affected by population density, whereas recruitment age variation appeared to be better explained by climatic conditions, with a surprising long‐term effect of climate. While population size explained 60% of the variation in juvenile survival, the average Southern Annular Mode over the five previous years explained 52% of variation in recruitment age.
4. In addition, although early‐life survival was consistently negatively affected by population size, the relationship between recruitment age and population size shifted from negative to positive over time from the 1970s to 2000s, showing that density dependence mechanisms can temporarily disappear.
5. Finally, we found that similar climatic conditions may affect individual performances in opposite ways according to the life stage of individuals. This result underlines the critical need to assess age‐specific functional responses to environmental variability to allow accurate demographic predictions. By revealing the poorly known demographic process of younger age classes, the results of this study improve our understanding of population dynamics of long‐lived marine species.

A lay summary is available for this article.

## Introduction

The impacts of global change are now well documented for several levels of biological organization from individual to ecosystem scales (Vitousek 1994; Walther *et al*. 2002; Parmesan 2006; Dillon, Wang & Huey 2010). At the population level, variation in demographic rates have been related to climatic and anthropogenic perturbations for diverse animal and plant populations (Coulson 2001; Grosbois *et al*. 2008; Inouye 2008; Barbraud *et al*. 2012). However, most of these studies focused on the vital rates of the adult component of populations (e.g. adult survival, fertility), whereas the demographic sensitivity of the immature component to climate perturbations (e.g. juvenile survival, recruitment) has been less widely investigated (Coulson 2001; Nevoux, Weimerskirch & Barbraud 2010; Dybala *et al*. 2013). Nevertheless, younger age classes are generally more sensitive to environmental variation (Gaillard *et al*. 2000; Oro *et al*. 2010; Pardo *et al*. 2013) owing to their inexperience and lower competitive abilities (Wunderle 1991; Sol *et al*. 1998). Thus, to fully understand the effects of climatic change on age‐structured populations and to be able to establish sound predictions from population models, the effects of environmental variability on the vital rates of the immature component of populations need to be considered. Because younger age classes represent a significant part of the population and account for a large contribution to the total reproductive value and demographic stochasticity (Sæther *et al*. 2013), early life stages are particularly important for long‐lived species.

Although there is an increasing need to predict the responses of populations to climate change for conservation purposes, investigating relationships between demographic processes and environmental conditions remains challenging for at least four reasons. First, such studies require long‐term datasets which are difficult to run and maintain (Clutton‐Brock & Sheldon 2010). Second, studies focusing on young individuals raise additional challenges since survival of early‐life stages is typically low (Newton 1989) and accompanied by a high dispersal probability (Clobert *et al*. 2001). Estimating early‐life survival under such constraints requires both large sample sizes and large scale monitoring over extended periods. Third, studying the relationships between population dynamics and environmental variation is often difficult owing to the simultaneous effects of different ecological factors (Gaillard *et al*. 2000; Constable *et al*. 2014). At the same time, studies based on the monitoring of wild populations cannot experimentally control environmental parameters. Intrinsic factors, such as density dependence, may also interact with external climatic constraints leading to complex ecological interactions (Coulson 2001). Finally, generalization of the results obtained from one population is often difficult since different populations of the same species may respond differently to the same environmental variation due to locally specific responses to environmental changes and complex ecological interactions (Walther *et al*. 2002; Constable *et al*. 2014).

Seabirds are frequent model species for biological population studies due to practical reasons, that is, they breed in large colonies distributed in discrete units and show high site fidelity. However, while there are evidences that climate change can affect their abundance (Croxall, Trathan & Murphy 2002; Weimerskirch *et al*. 2003), the underlying demographic processes at the individual scale remain often unknown, in particular for the immature component. Juvenile traits have been much less studied than adult vital rates (Jenouvrier 2013; Oro *et al*. 2014). Since young individuals have an important impact on demographic variability in long‐lived species (Sæther *et al*. 2013), understanding causal demographic mechanisms at the individual scale including all life stages is essential to predict future population trends in response to climate change. Seabirds are today one of the most threatened group among birds (IUCN 2016), and the improvement of our knowledge on juvenile vital rates was defined as a priority for seabird research (Lewison *et al*. 2012).

In this article, we investigated the climatic effects on immature demographic parameters in a long‐lived seabird, the wandering albatross *Diomedea exulans*. An individual‐based study of 9685 birds, conducted on the Crozet Islands (Southern Indian Ocean) since 1965, allowed us to estimate early‐life survival and age of first reproduction. The climate in the Southern Ocean is changing rapidly (Pendlebury & Barnes‐Keoghan 2007) impacting seabirds and marine mammal populations (Weimerskirch *et al*. 2003; Constable *et al*. 2014). Previous studies have investigated climate effects on adult performances in the wandering albatross (Rolland, Weimerskirch & Barbraud 2010; Weimerskirch *et al*. 2012). However, very few focused on immature individuals and relationships with climatic factors remain equivocal (Fay *et al*. 2015a,b). Using capture‐mark‐recapture modelling, we investigated the impact of climate and population density on early‐life demographic traits (survival and recruitment age) testing for both linear and quadratic relationships and interactions between climate and population size. Our models controlled for age, sex and density dependence since all these factors are important to understand demographic processes of immature wandering albatrosses (Fay *et al*. 2015a).

## Materials and methods

### Study species and site

The wandering albatross population of Possession Island in the Crozet Islands (46°S; 52°E), southern Indian Ocean, has been monitored from 1960. Annual mark‐recapture studies started in 1965. From 1965, chicks were ringed each year with a stainless steel band just before fledging. The breeding cycle of this quasi‐biennial species lasts almost 1 year, with pair formation in December, laying in early January, hatching of the egg in April and fledging of the chick in November (Tickell 2000). Clutch size is limited to one egg without replacement laying. There is no post‐fledging care and the fledglings leave the colony alone at the age of 9 months, remaining at sea continuously for the following 2 to 7 years (Weimerskirch 1992). Juvenile (aged 1–2 years) wandering albatrosses remain in the tropical and sub‐tropical waters of the Indian Ocean (Weimerskirch *et al*. 2014). After 2 years at sea, their range shifts southward, and young immature birds start to return to their natal colony before starting to breed when 6 years old at the earliest (Weimerskirch 1992). This population showed important changes in abundance over the study period. From 500 breeding pairs in the 1960s, it declined steeply in the 1970s down to 240 pairs in the mid‐1980s, and then increased progressively to 380 pairs in the 2000s with a subsequent decline thereafter (Delord *et al*. 2008).

### Field methodology

From early to mid‐December, pre‐breeding adults were monitored across the whole island. From mid‐January (just after egg laying) to mid‐February at least three visits were made every 10 days to identify breeding pairs and their status. All new individuals were ringed with a uniquely numbered stainless steel‐band. In mid‐April, June and August, nests were checked and the chick status recorded (alive/dead). During all visits, non‐breeding individuals (mainly immatures) were searched for and their identity determined (from ring number) when possible. From mid‐September to mid‐October fledglings were ringed. Chicks that died on the colony between ringing and fledgling were noted during the first checks of the following breeding cycle and were excluded from our dataset (0·3% of all individuals). Sex assignment was carried out as described in Appendix S1 (Supporting Information).

### General model

**Figure 1:**
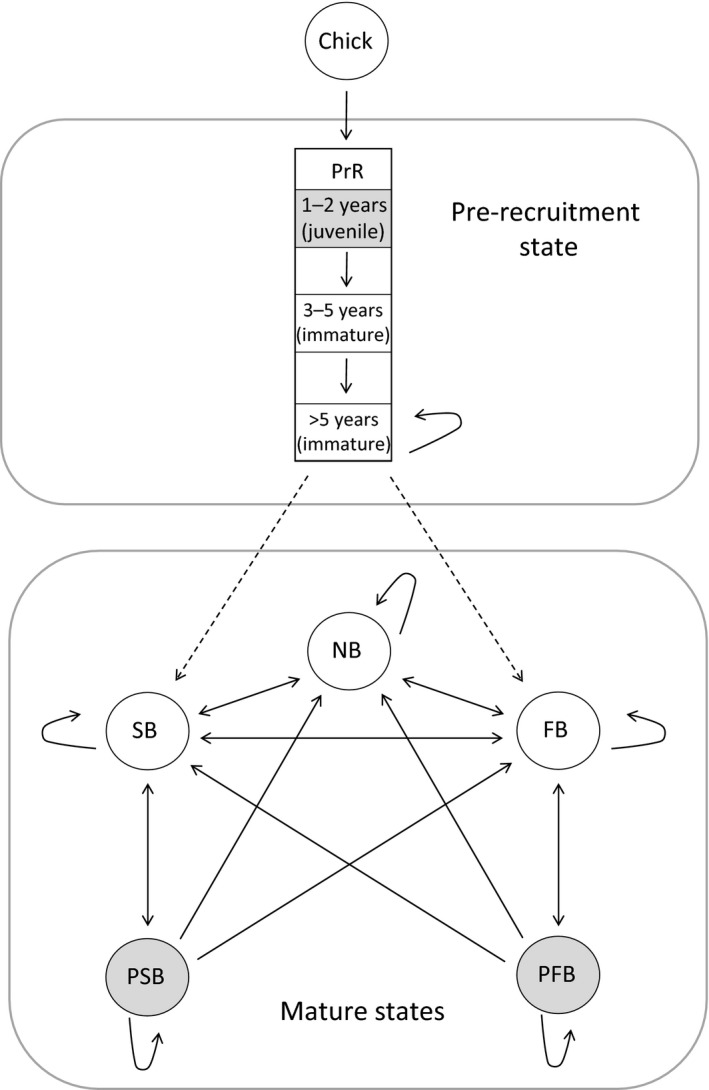
Life cycle graph representing transitions between observable (white) or unobservable states (grey). All birds are ringed as chicks, thus individuals start in the Pre‐recruitment state (PrR). After fledging, all birds remain at least 2 years continuously at sea (i.e. juvenile stage). Pre‐recruitment state becomes observable from 3 years old when birds start to return to the colony and are now considered as immature. From 6 years old, birds can pass into the breeding group of the population though recruitment represented by dashed arrows. Then mature birds irrespectively of age can transit between mature states: successful breeder (SB), failed breeder (FB), recruited non‐breeder (NB), post successful breeder (PSB) and post‐failed breeder (PFB).

Individual encounter histories were modelled using a multi‐event approach. The model comprised seven states consisting of one immature state, five adult states and the state dead (Fig. 1), and five events. To consider individuals during the period of immaturity, we defined the Pre‐Recruitment state (PrR) after which immature birds can recruit, i.e. lay an egg for the first time into the breeding population. Adult birds could transit towards Successful Breeder state (SB), when the chick fledged, Failed Breeder state (FB), when the egg or chick was lost before fledging, or recruited Non Breeder state (NB), when individuals that have recruited in the population (i.e. bred at least once) were observed as non‐breeders at the colony. To model the sabbatical years spent continuously at sea after reproduction, we added two unobservable states (Lebreton & Pradel 2002), corresponding to the two previous breeding states defined: Post‐successful Breeder (PSB) and Post‐failed Breeder (PFB). Thus, adults that are at sea (i.e. not at colonies for a whole year) are distinguished based on their most recent breeding state last time they were at a colony. In our study, state assignment was not always certain since between 1966 and 1986, state was unknown for a number of breeders; some individuals were classified as breeders but the success or failure was not always ascertained. Multi‐event models allowed us to deal with state uncertainty by assessing the likelihood of an individual state given the events (i.e. observations) (Pradel 2005). We considered five events, i.e. five types of observation in the field, 0 = ‘not observed’, 1 = ‘seen as non‐breeder’, 2 = ‘seen as a failed breeder’, 3 = ‘seen as a successful breeder’, 4 = ‘seen as a breeder but successful status not ascertained’. Details of the parametrization with the biological constraints applied can be found in Appendix S2. Capture–recapture modeling does not allow the direct estimation of the average age of first reproduction for each year. Thus, variation in the age of first reproduction was estimated indirectly through the probability of early recruitment (ψ^early^). Early recruitment was defined as the first reproduction occurring ≤8 years old females and 9 years old for males, corresponding approximately to the average age of first reproduction for both sexes (see Fig. S2).

### Density dependence and climatic variables

Juvenile survival was modelled as a function of population size since a recent study revealed that this parameter was strongly density dependent (Fay *et al*. 2015a, b). For the recruitment process, the relationship with population size was more equivocal. Weimerskirch, Brothers & Jouventin (1997) reported decreasing age at first reproduction simultaneously to declining breeding population size, whereas a recent study, including additional years, concluded that the recruitment process was currently free from a density effect (Fay *et al*. 2015a, b). Considered together, these studies suggest that a shift occurred in this population regarding density dependence effects on the recruitment process. We thus investigated the relationship between recruitment age and population size, using 25 successive 10‐year windows moving along the time series by a 1‐year step (*t*_1_ to *t*_10_, *t*_2_ to *t*_11_,…, *t*_25_ to *t*_35_, Durant *et al*. 2004). Obtaining the same correlation between recruitment age and population size for all windows would support a single linear relationship over the whole study period. Otherwise, nonlinear processes and shifts in relationships may be supported. We used cohort‐specific recruitment estimates to have the largest temporal windows available. To model population density effects on early‐life survival and recruitment, we used the number of breeding pairs observed annually at Possession Island as a covariate.

Our selected climate covariates included a large‐scale climate index, the Southern Annular Mode (SAM) and a local climatic variable calculated over age‐specific home ranges at sea (Fig. S1), the Sea Surface Temperature Anomaly (SSTA). SAM is the leading mode of atmospheric circulation variability in the Southern Hemisphere affecting the Southern Ocean (Marshall 2003). This climatic index may affect albatrosses both directly through wind speed (Weimerskirch *et al*. 2012), and indirectly, affecting primary productivity through large‐scale impact on ocean mixed layer depth (Sallée, Speer & Rintoul 2010). SAM data were selected from the online database of the British Antarctic Survey (http://www.nerc-bas.ac.uk/icd/gjma/sam.html). At a regional scale, SSTA may determine the development and productivity of the whole trophic web, and hence may be used as a proxy of oceanographic conditions. SSTA have been related to demographic parameters for many seabird species in the Southern Ocean and elsewhere, probably through indirect mechanisms such as the abundance of food resources (Sandvik *et al*. 2005; Barbraud *et al*. 2012). SSTA data were obtained from the IRI Data Library http:/iridl.ldeo.columbia.eduSOURCES/.NOAA/.NCEP/.EMC/.CMB/.GLOBAL/.Reyn_SmithOIv2/.monthly/.ssta/

First, we assessed the impact of selected covariates on pre‐recruitment survival considering their annual averages the previous year. We included the natal climatic conditions for juvenile survival since this parameter is expected to be affected by climatic conditions both during chick stage and after fledging. A recent study suggested that high natal SSTA (i.e. SSTA during the chick rearing period) on parental foraging grounds had negative effects on post‐fledging survival (Fay *et al*. 2015a, b). This effect is expected to occur through decreasing paternal investment in chick rearing when facing poor environmental conditions leading to a negative impact on chick condition at fledging (McMahon & Burton 2005). In the absence of reliable data on the body condition of chicks, we tested this hypothesis indirectly, assessing the effect of SSTA on parental foraging grounds by distinguishing the early rearing stage (April–July) from the late rearing stage (August–November) when chicks constituted their fat reserves (Reid, Prince & Croxall 2000). As parental investment and foraging areas are sex‐dependent in the wandering albatross (Weimerskirch, Barbraud & Lys 2000), we distinguished SSTA on the males’ (SSTAma) and females’ foraging grounds (SSTAfe).

Knowing that access to reproduction may take many years in this species (Weimerskirch 1992), we investigated the effect of past climatic conditions on recruitment. We first assessed the effect of a climatic covariate on recruitment age by considering its annual average the previous year as for the survival parameter. Then, we assessed the longer term effect of this covariate by considering its average value calculated for the previous 2 years. If this new covariate improved our model, we tested a third covariate by considering its average value calculated for the three previous years, and so on until the percentage of variation explained by the climatic variable stabilized. Once this stage was reached, integrating additional years did not explain more residual variation, and we considered that the demographic parameter varied independently of these past climatic conditions.

All environmental variables were normalized prior to analysis. We fitted the logistic model: logit(Φ) = β_0_ + β_1_ × *x*_*n*,_ where Φ is a demographic parameter, β_0_ is an intercept parameter, β_1_ is a slope parameter and *x*_*n*_ is the covariate *x* the year *n*. Significant relationships were assessed by an analysis of deviance test with a Fisher–Snedecor distribution (ANODEV; Grosbois *et al*. 2008). The percentage of variation that was explained by a covariate (*r* ^2^) was estimated as: *r* ^2 ^= [(Dev(*F*_cst_) − Dev(*F*_cov_)]/[Dev(*F*_cst_) − Dev(*F*_*t*_)] (Skalski 1996). To limit the number of tests, we investigated quadratic relationships and interaction effects between climatic variables and population size only for the covariates having received a minimum level of support (*P*‐value ≤ 0·10).

## Results

Goodness‐of‐fit tests (χ^2^ = 1003·4, d.f. = 749, *P* < 0·001 for females and χ^2^ = 1263·8, d.f. = 898, *P* < 0·001 for males) indicated that the general JMV model did not fit the data correctly (see Table S1). We thus used a variance inflation factor (ĉ = 1·37) for model selection. Results indicated that early‐life survival and recruitment processes were strongly influenced by environmental variability and population density. We found that population size constrained both early‐life survival and recruitment age, although in different ways.

**Figure 2:**
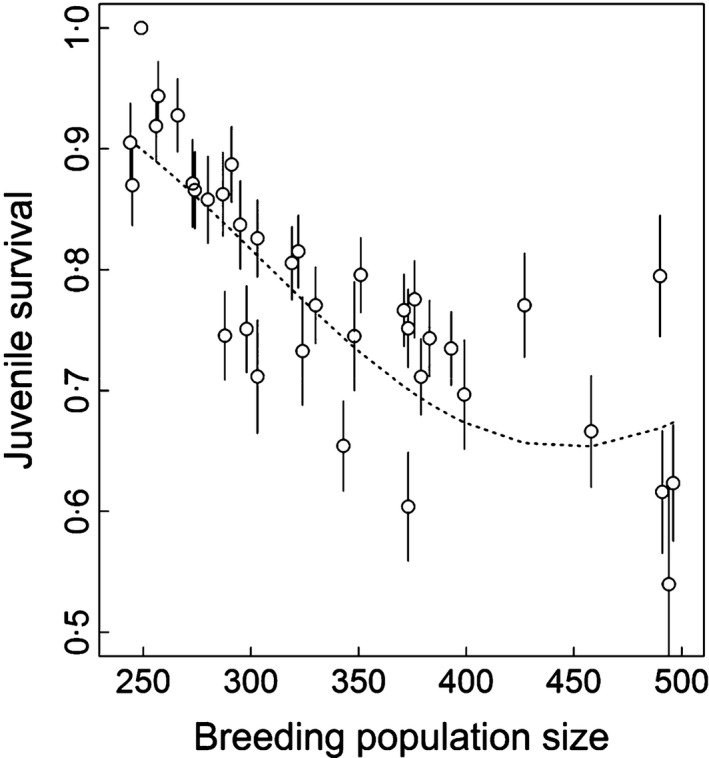
Relationship between juvenile survival and breeding population size for the wandering albatross population of Crozet. Estimates of juvenile survival obtained from the time‐dependent model (open circles ±SE) and juvenile survival modelled as a function of breeding population size (dotted line, Table 1 Model 2, *P*_Anodev_ = 0·01).

**Figure 3:**
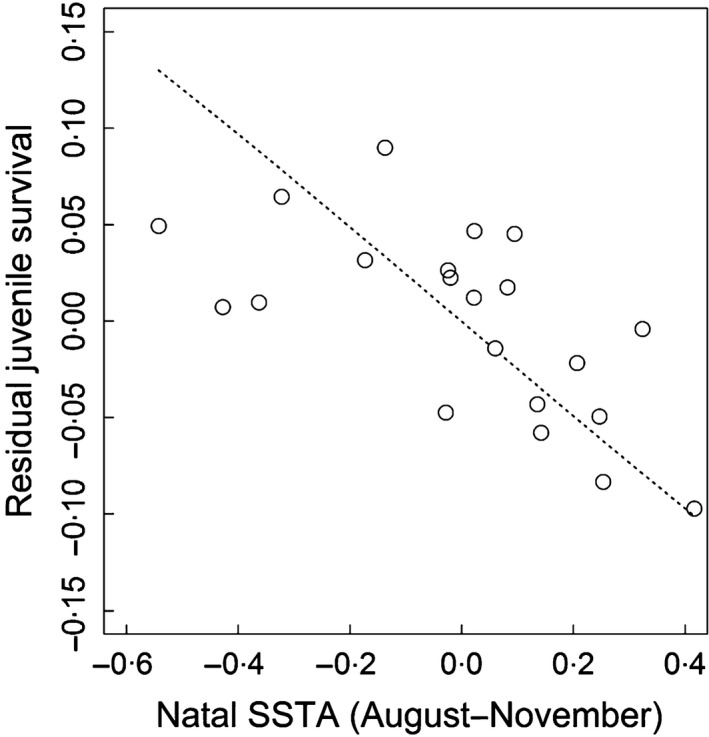
Residual juvenile survival (open circles) modelled as a function of natal Sea Surface Temperature Anomaly (SSTA) on male foraging grounds during the late rearing stage (August–November) for the wandering albatross population of Crozet (Dotted line, Table 1 Model 7, *P*_Anodev_ = 0·03). Residual survival estimates were calculated as the difference between survival estimates obtained from the time‐dependent model and survival estimates obtained from the model where survival was modelled as a function of breeding population size.

Model selection confirmed the relationship between juvenile survival and population size, with a better fit obtained with a quadratic relationship that explained 60% of the total variance (Table 1, *F*test_cst/co/*t*_ = 4·98, *P*‐value < 0·01 and Fig. 2). Controlling for the effect of abundance, we found an additive linear negative effect of natal SSTA on paternal foraging ground, but not on maternal, during the late stage of chick rearing (Table 1, *F*test_cst/co/*t*_ = 4·24, *P*‐value = 0·03), that explained 31% of the residual variance (Fig. 3). No relationship was supported for SSTA during the early stage of chick rearing (Table 1, *F*test_cst/co/*t*_ = 0·30, *P*‐value = 0·74). Model selection did not support an effect of natal SAM condition on juvenile survival or an effect of SAM and SSTA in the first year after fledging. We found no support for an interaction of natal SSTA with breeding population size. In contrast to juveniles, immature survival was not affected by population size (Table 1, *P*‐value = 0·76), and we did not find evidence for an impact of climatic conditions on this parameter.

**Table 1:** Testing for the effects of covariates on juvenile and immature survival for wandering albatrosses from Crozet Island from 1965 to 2012

No.	Pre‐recruitment survival	*Q*_Dev_	*F*	*P*‐value	*r* ^2^	Slope [95% CI]
	1–2 years old class					
**1**	***N***	**79817·20**	**17·10**	**<0·001**	**0·49**	**−0·47 [−0·56; −0·37]**
**2**	***N*** **_qua**	**79793·02**	**4·98**	**0·01**	**0·60**	
3	*N*_qua + SAM	79789·99	0·65	0·53	0·03	+0·07 [**−**0·01; +0·15]
4	*N*_qua + SSTA	79784·22	0·12	0·89	0·01	**−**0·04 [**−**0·16; +0·08]
5	*N*_qua + SAMnat	79785·12	1·79	0·18	0·09	+0·11 [+0·03; +0·19]
6	*N*_qua + SSTAmaAprJul	79783·45	0·30	0·74	0·03	**−**0·06 [**−**0·16; +0·05]
**7**	***N*** **_qua + SSTAmaAugNov**	**79771·78**	**4·24**	**0·03**	**0·31**	**−0·25 [−0·39; −0·11]**
8	*N*_qua + SSTAmaAugNov_qua	79769·95	0·64	0·54	0·35	
9	*N*_qua + SSTAfeAprJul	79784·60	0·03	0·97	<0·01	**−**0·01 [**−**0·10; +0·08]
10	*N*_qua + SSTAfeAugNov	79784·46	0·06	0·94	0·01	**−**0·02 [**−**0·14; +0·09]
11	*N*_qua. SSTAmaAugNov	79773·94	0·41	0·67	0·34	
	3–8 years old class					
12	*N*	79675·77	0·28	0·76	0·02	**−**0·04 [**−**0·12; +0·05]
13	SAM	79674·29	0·88	0·42	0·05	**−**0·13 [**−**0·31; +0·04]
14	SSTA	79667·74	1·66	0·19	0·11	**−**0·17 [**−**0·30; **−**0·04]

Results include the relative deviance corrected by the overdispersion factor (*Q*_Dev_), the statistic *F*test_cst/co/t_ testing the null hypothesis that the focal climatic covariate has no effect on survival, the percentage of variation or residual variation explained by the covariates or additive covariate respectively (*r* ^2^) and the 95% CI of the slope for linear relationships. Symbols ‘.’, and ‘+’ indicate interactive and additive effects respectively. All covariates were standardized. Models with statistically significant covariate effects at the level of 5% are in bold characters. SAM, Southern Annular Mode; SSTA, Sea Surface Temperature Anomaly.

**Figure 4:**
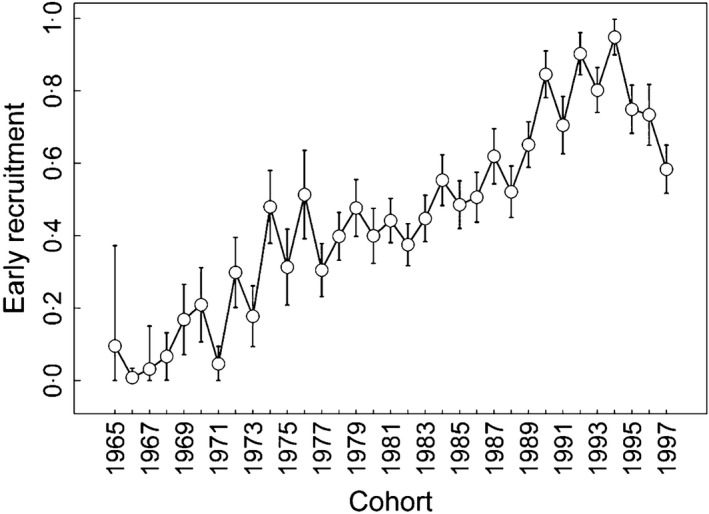
Cohort‐specific early‐recruitment probability (**±** SE) of the wandering albatross population of Crozet. Early recruitment was defined as first reproduction occurring before or equal to 8 years old for females and 9 years old for males (see Fig. S2).

**Figure 5:**
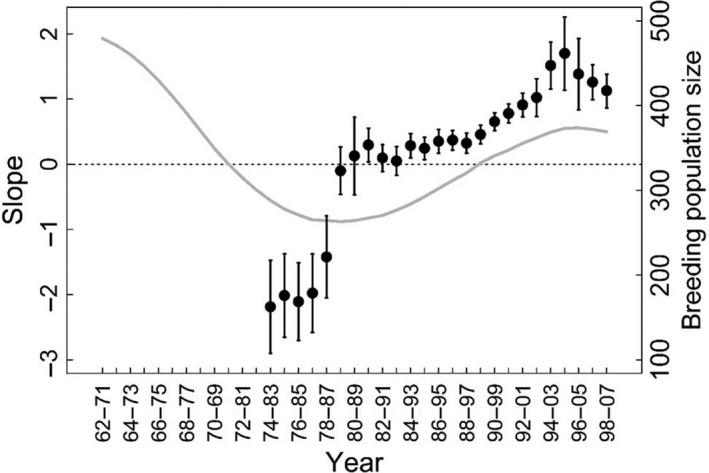
Slope of the relationships over the successive 10‐year windows between early recruitment probability and breeding population size for the wandering albatross population of Crozet. Values of the slopes of the relationships (filled circles ±SE) and 10‐year moving average breeding population trend (grey line).

**Figure 6:**
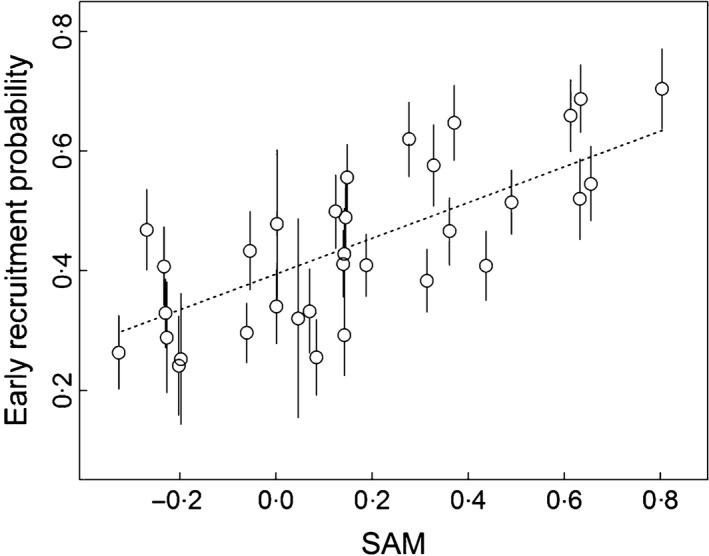
Relationship between early recruitment probability and SAM, Southern Annular Mode (SAM) averaged over the five previous years for the wandering albatross population of Crozet. Estimates of early recruitment obtained from the time‐dependent model (open circles ±SE) and early recruitment probability modelled as a function of standardized SAM averaged over the five previous years (dotted line, Table 3 Model 9, *P*_Anodev_ < 0·001).

The probability of early‐recruitment was highly variable between years (Table 2, M2 vs. M3, ΔQAIC = 27·2). These variations did not differ between sexes (Table 2, M1 vs. M2, ΔQAIC = **−**20). Considering cohort‐specific early‐recruitment probability, we found that recruitment age decreased continuously during the study period until the cohort born in 1994 (Fig. 4). We found a shift in the relationship between early‐recruitment probability and population size. During the 1970s when the breeding population size decreased from 500 to 250 pairs, we found a negative relationship between recruitment age and population size (Fig. 5). However, between the mid‐1980s and the early 2000s when the population was recovering slowly to stabilize at around 380 pairs, recruitment age continued to decrease. Consequently, early recruitment was positively related to population size, suggesting that density dependence was no longer occurring. Additionally, recruitment age was strongly related to climatic factors. Results support a positive linear effect of SAM on early‐recruitment probability with a long‐term effect of this large‐scale climate index. Indeed, model fit increased continuously until the integration of the SAM value over the five previous years (Table 3), which explained 52% of the variation in recruitment age (Fig. 6). SAM presented a linear temporal trend (slope = 0·04, *P*‐value = 0·02) as well as early‐recruitment probability over the study period (Fig. 4). To check for the robustness of our result, we removed the linear trend of SAM using the residual regression technique and reanalysed the relationship between early‐recruitment and this detrended climatic covariate. We found again strong support for a long term effect of SAM on recruitment process (*F*test_cst/co/*t *_= 7·24, *P*‐value = 0·003). Focusing on local climatic covariates, model selection supported a positive linear effect of SSTA (Table 3). Long‐term effects of SSTA appeared weaker in this case receiving support only for the two previous years (Table 3). No interaction was supported between climatic covariates and breeding population size.

**Table 2:** Recruitment age (early vs. late recruitment) modelling as a function of sex and time (*t*) of the wandering albatross population of Crozet Island from 1965 to 2012

No.	Recruitment age	*k*	*Q*_Dev_	*Q*_AIC_
**1**	**sex + ** ***t***	**154**	**79562·21**	**79870·21**
2	sex.*t*	186	79518·28	79890·28
3	sex	122	79673·48	79917·48
4	cst	121	79677·51	79919·51

Results of model selection include: number of mathematical parameters (*k*), the relative deviance corrected by the overdispersion factor (*Q*_Dev_) and Akaike Information Criterion value corrected by the overdispersion factor (*Q*_AIC_). Symbols ‘.’, and ‘+’ indicate interactive and additive effects respectively. The best supported model is in bold characters.

**Table 3:** Testing for the effects of covariates on recruitment age for wandering albatrosses from Crozet Island from 1965 to 2012

No.	Pre‐recruitment survival	*Q*_Dev_	*F*	*P*‐value	*r* ^2^	Slope
	Single covariate model					
1	SAM(*t* **−**1)	79655·19	3·05	0·06	0·16	+0·22 [+0·12; +0·33]
2	SAM(*t* **−**1)_qua	79654·47	0·12	0·89	0·17	
**3**	**SAM(** ***t*** **−2)**	**79647·38**	**4·75**	**0·02**	**0·23**	**+0·25 [+0·15; +0·35]**
4	SAM(*t* **−**2)_qua	79646·42	0·17	0·84	0·24	
**5**	**SAM(** ***t*** **−3)**	**79631·52**	**9·38**	**<0·001**	**0·38**	**+0·33 [+0·22; +0·43]**
6	SAM(*t* **−**3)_qua	79630·48	0·24	0·79	0·39	
**7**	**SAM(** ***t*** **−4)**	**79624·02**	**12·40**	**<0·001**	**0·44**	**+0·35 [+0·25; +0·45]**
8	SAM(*t* **−**4)_qua	79623·23	0·20	0·82	0·45	
**9**	**SAM(** ***t*** **−5)**	**79615·54**	**16·84**	**<0·001**	**0·52**	**+0·38 [+0·28; +0·48]**
10	SAM(*t* **−**5)_qua	79613·93	0·48	0·62	0·54	
**11**	**SAM(** ***t*** **−6)**	**79614·34**	**17·58**	**<0·001**	**0·53**	**+0·38 [+0·28; +0·49]**
12	SAM(*t* **−**6)_qua	79612·01	0·73	0·49	0·55	
**13**	**SSTA(** ***t*** **−1)**	**79648·94**	**4·22**	**0·02**	**0·22**	**+0·25 [+0·14; +0·36]**
14	SSTA(*t* **−**1)_qua	79648·69	0·04	0·96	0·23	
**15**	**SSTA(** ***t*** **−2)**	**79638·23**	**7·06**	**0·003**	**0·33**	**+0·30 [+0·20; +0·40]**
16	SSTA(*t* **−**2)_qua	79636·56	0·35	0·71	0·34	
**17**	**SSTA(** ***t*** **−3)**	**79638·87**	**6·86**	**0·04**	**0·32**	**+0·30 [+0·19; +0·40]**
18	SSTA(*t* **−**3)_qua	79632·74	1·36	0·26	0·38	
	Multiple covariate model					
19	SAM(*t* **−**5) + SSTA(*t* **−**2)	79612·06	1·19	0·32	0·54	
20	SAM(*t* **−**5). N	79596·73	1·40	0·26	0·69	
21	SSTA(*t* **−**2). N	79595·67	0·01	1	0·74	

Results include the relative deviance corrected by the overdispersion factor (*Q*_Dev_), the statistic *F*test_cst/co/*t*_ testing the null hypothesis that the focal climatic covariate has no effect on recruitment age, the percentage of variation explained by the covariates (*r* ^2^) and the 95% CI of the slope for linear relationships. Symbols ‘.’, and ‘+’ indicate interactive and additive effects respectively. All covariates were standardized. Models with statistically significant covariate effects at the level of 5% are in bold characters. SAM, Southern Annular Mode; SSTA, Sea Surface Temperature Anomaly.

## Discussion

In this study, we provided evidence that climate and population size affected both the survival and recruitment age of young individuals but in different ways according to the trait. We found that early‐life survival was mainly affected by population density whereas recruitment age variation appeared better explained by climatic conditions with a long‐term effect of climate. Furthermore, results suggested that similar climatic conditions had opposite effects on individual performance according to the life stage.

### Density dependence

While population density as a regulating process of populations has often been mentioned in terrestrial environments (Bonenfant *et al*. 2009), it has been less commonly reported in marine species, especially for seabirds that frequently range over vast oceanic areas (Lewis *et al*. 2001). In this study, we showed that both early‐life survival and recruitment age could be constrained by population density in wandering albatrosses. Density effects seemed higher for juvenile survival than for recruitment age, supporting Eberhardt's idea that vital rates have different sensitivities to changes in population density (Eberhardt 2002).

A negative effect of population size on early‐life survival was clearly established in wandering albatrosses (Fay *et al*. 2015a, b), but we showed that recruitment age could be affected as well. Earlier studies for the same study population reported decreasing age at first reproduction in the early 1970s associated with decreasing population size, and concluded a density‐dependent effect (Weimerskirch & Jouventin 1987; Weimerskirch, Brothers & Jouventin 1997). However, a recent study based on a different, but larger time‐window from the 1980s to the 2010s suggested contrasting patterns with overall positive relationships between population size and recruitment (Fay *et al*. 2015a, b). Here, by investigating the effect of population size on recruitment age over the whole study period, we showed that the effect of population density on this parameter shifted over the course of time. In the 1970s, recruitment age decreased simultaneously to decreasing breeding population size, as found for other long‐lived species. In these organisms, young individuals delayed reproduction beyond the minimum age of sexual maturity, waiting for vacant breeding sites or territories (Sæther, Engen & Matthysen 2002). This resulted in a queuing process that allowed massive recruitment when an important adult mortality event occurred (Pradel *et al*. 1997; Votier *et al*. 2008). Such mechanisms, known as compensatory recruitment, could be associated with decreasing recruitment age (Reid *et al*. 2003; Ferrer, Otalora & GarcÍa‐Ruiz 2004). In our study population, a similar queuing process may explain decreasing age at first reproduction occurring after the population crash in the 1960s, when adult survival decreased substantially (Weimerskirch & Jouventin 1987). However, for wandering albatross, which breeds in loose aggregations on the grassy plateaus of oceanic islands, nests or territories are probably not limiting (Tickell 2000). Rather, in this species, recruitment seems mainly constrained by body condition since immature individuals must attain a sufficient condition to be able to recruit into the population (Weimerskirch 1992). With decreasing population size, immature birds may have been able to attain this condition earlier because of a reduction in competition at sea. However, after a phase of stabilization during the 1980s, recruitment age continued to decrease while the population recovered progressively from the mid‐1990s to the early 2000s, resulting in a positive relationship between early recruitment probability and breeding population size. This result was not expected since, other things being equal, the age at first reproduction in long‐lived species usually increases with population size (Gaillard *et al*. 2000; Reid *et al*. 2003; Ferrer, Otalora & GarcÍa‐Ruiz 2004). However, the effect of population density could be nonlinear and demographic traits could be impacted by density only at high population levels (Strong 1986). Thus, we speculate that in recent decades, population size remained below the threshold for which density‐dependent recruitment may occur, since the population had not yet recovered its original size of the 1960s. With the effect of density dependence being relaxed, this albatross population showed a positive relationship between recruitment and breeding population size typical of short‐lived species. In short‐lived species, survival, and not territory occupancy, is the main limiting factor restricting recruitment rate, and favourable environmental conditions increase both the return rate of adults and the number of recruits (Sæther, Engen & Matthysen 2002). In this context, decreasing recruitment age since the 1990s may reflect favourable environmental conditions allowing birds to reach a sufficient body condition for breeding at younger ages (Weimerskirch 1992; Weimerskirch *et al*. 2012).

Alternatively, although non‐exclusive, decreasing age at first reproduction with increasing population size could be interpreted as an Allee effect (Allee 1931), that is, a positive density effect at low population level. At low population densities, mating processes can be disturbed leading to positive relationships between population size and recruitment rates (McCarthy 1997; Angulo *et al*. 2007). Long‐lived species adopting monogamous breeding systems with high mate fidelity such as the wandering albatross (Bried, Pontier & Jouventin 2003) could be particularly sensitive to the Allee effect.

### Climate effect

We found that both early‐life survival and recruitment age were strongly affected by climatic variations. Much of the juvenile survival variation was explained by natal conditions, with a negative effect of SSTA on paternal foraging grounds. Such natal effects may be mediated by parental investment. In long‐lived species, breeders facing poor environmental conditions are expected to decrease their breeding investment to protect their own survival, resulting in poor chick condition at independence and lower survival in early‐life (McMahon & Burton 2005). Consequently, the negative effect of natal SSTA was only supported during the late stage of chick rearing when young accumulated fat stores before fledging (Reid, Prince & Croxall 2000). Climatic conditions on maternal foraging grounds were not related to juvenile survival whatever the rearing stage. This result confirms previous studies suggesting that paternal characteristics in wandering albatrosses have a major importance for post‐fledging juvenile performances due to the higher investment of the male parent during chick provisioning (Fay *et al*. 2015a, b, 2016).

Recruitment age appeared to be highly variable in relation to climatic conditions. In particular, we found a positive relationship between SAM and early recruitment, with an unexpected long‐term effect of this climatic variable. Although little information is available to understand the underlying proximate mechanisms linking SAM to immature albatross demography, this long‐term climatic effect on recruitment age may be related to the progressive increase in weight observed in this species through immaturity (Weimerskirch 1992). In long‐lived species, the body condition is an important factor determining whether a young individual engages in reproduction or not (Martin & Festa‐Bianchet 2012). Bearing in mind that climatic effects on seabird demography are usually mediated by food availability (Ainley, Sydeman & Norton 1995; Durant, Anker‐Nilssen & Stenseth 2003; Bost *et al*. 2015), environmental conditions encountered several years before the first reproduction could impact individuals’ growth trajectories by affecting the time at which an individual reaches the body condition required to breed (Weimerskirch 1992).

Increasing sea surface temperature had a positive effect on the recruitment process, which is in contrast to results reported for juvenile survival. This suggests that similar climatic conditions may lead to different demographic responses according to the life stage considered. We suggest that these surprising results could be explained by the age‐specific spatial segregation observed in our study population. When ageing, wandering albatrosses progressively shift their foraging areas southward from sub‐tropical to sub‐antarctic water (Weimerskirch *et al*. 2014). The Southern Ocean is highly heterogeneous with many sub‐systems that do not respond equally to climatic variability (Tréguer & Jacques 1992; Lovenduski 2005). Relationships between SST and primary productivity seem heterogeneous between locations (Fauchereau *et al*. 2011), and lower temperatures may limit phytoplankton growth rates in some areas (Reay *et al*. 2001). In Antarctic and sub‐tropical zones, SST increases are usually negatively related to demographic performances, suggesting that in these areas increasing SST decreases food availability (Barbraud & Weimerskirch 2001; Weimerskirch, Zimmermann & Prince 2001; Jenouvrier, Barbraud & Weimerskirch 2003; Beauplet *et al*. 2005). In contrast, in the sub‐antarctic zone, where adult and immature wandering albatrosses are found before recruitment, the negative effects of warm waters seem less clear (Fauchereau *et al*. 2011; Takao *et al*. 2012). In this area, studies reported both positive (Nevoux, Weimerskirch & Barbraud 2007; Rolland *et al*. 2009; Oosthuizen *et al*. 2015) and negative (Guinet *et al*. 1998; Bost *et al*. 2015) relationships between SST and top predator demography. The latitude at which the species forage within the sub‐antarctic zone seems critical to the sign of these relationships. Species foraging in the north of this area seem to have better demographic performances than those foraging to the south when facing positive SSTs (Inchausti *et al*. 2003). Consistently, we found that adult male wandering albatrosses foraging in the south of the sub‐antarctic zone, close to the Polar Front, were negatively affected by positive SSTs, whereas immature individuals staying at lower latitudes responded positively to the same climatic conditions (Weimerskirch *et al*. 2014). Thus, we suggest that age‐specific demographic responses to SSTs observed in wandering albatrosses may be caused by the large home range of this population in relation to local oceanic responses to climatic variations. Such result shows that it is essential to consider age effects to understand population responses to climate change, since similar climatic conditions may have opposite effects on individual performances according to the life stage considered (Dybala *et al*. 2013; Pardo *et al*. 2013; Radchuk, Turlure & Schtickzelle 2013).

In this study, we have shown that early‐life survival and recruitment could be affected by population density and climatic variation although in different ways according to the life stages. Such results highlight the need to assess age‐specific functional responses to environmental variability to allow accurate demographic prediction. Furthermore, we found a shift in the effect of density dependence on recruitment age. In the context of global decreasing seabird abundances, this suggests that density‐dependent mechanisms can temporarily disappear, especially when decreasing population size is not related to food depletion. In such circumstances, while density‐dependent processes could be less evident, they still need to be considered when establishing long‐term population size projections. More importantly, in the context of climate change and rising temperatures, shifts in frontal zones and the forcing of wind speed in the southern ocean, the response of juvenile, immature and adult albatrosses will be different. Being able to estimate the respective influences of environmental parameters in different water masses on the survival of age classes as we did in our study will be critical to be able to make robust predictions on the impact of climate change on marine predators.

## Data accessibility

Dataset is available from the Dryad Digital Repository https://doi.org/10.5061/dryad.p62h7 (Fay *et al*. 2015b).

## Supporting information


**Lay Summary**

**Appendix S1.** Sex assignment.
**Appendix S2.** Parametrization of the general model and biological constraints.
**Fig. S1.** Distribution area from which SSTA values were extracted.
**Fig. S2.** Cumulated probability to be recruited.
**Table S1.** GOF tests results.
