# Supplementary material for: Contrasting effects of climate and population density over time and life stages in a long‐lived seabird
Source: Funct Ecol. 2017 Apr 3;31(6):1275–84. doi: 10.1111/1365-2435.12831 (PMC5518763; doi:10.1111/1365-2435.12831)
Supplement: Supplementary file 1 — Lay Summary [file FEC-31-1275-s001.pdf]

Functional Ecology

British Ecological Society

Contrasting effects of climate and population density over time and life-stages in a long-lived seabird

Rémi Fay, Christophe Barbraud, Karine Delord and Henri Weimerskirch

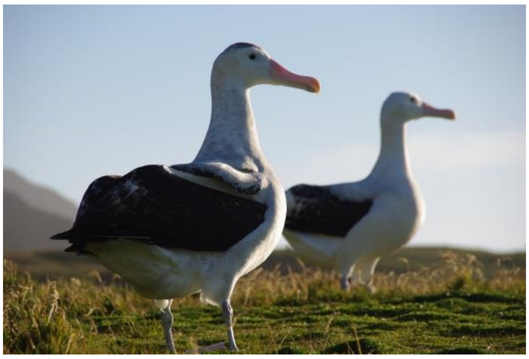

Photo credit: Aurélien Prudor

Demographic processes of wild populations are affected by environmental variability. Such functional relationships have been extensively studied for many organisms but mainly through adult vital rates (e.g. adult survival and fertility). So far, early-life stages have strikingly been less studied because of the inherent difficulties in tracking the fate of young individuals. Yet, young organisms are expected to be more sensitive to environmental stochasticity owing to their inexperience and lower competitive abilities. Hence, this lack of information on the juvenile compartment of populations currently constrains our ability to fully understand population dynamics.

In this study, we investigated the effect of climate and population density on early-life demographic parameters of a long-lived seabird, the wandering albatross. We provided evidence that climate and population size affected both survival and age at first reproduction of young individuals, but in different ways according to the trait. In particular, although both early-life demographic parameters are affected by population density, we found that survival was more strongly impacted by density than age at first reproduction, showing different sensitivities to changes in population density. We also found a shift in the effect of population density on age at first reproduction, suggesting that density dependence mechanisms can temporarily disappear. In the context of globally decreasing seabird abundance, density dependent processes could be less evident but still essential to consider for long term population size projections.

With regard to environmental conditions, we showed that increasing sea surface temperature had a positive effect on young individuals becoming reproductive at a younger age, while at the same time, it had a negative impact on juvenile survival. We suggest that age-specific demographic responses to sea surface temperature observed in wandering albatrosses may be caused by the age-specific at sea distribution and thus local oceanic responses to climatic variation. Such results show that it is essential to consider age effects to understand population responses to climate change, since similar climatic conditions may have opposite effects on individual performances according to the life stage considered.

Such results are critical for our ability to make robust predictions on the impact of climate change on marine predators such as albatrosses.
