# Supplementary material for: Contrasting effects of climate and population density over time and life stages in a long‐lived seabird
Source: Funct Ecol. 2017 Apr 3;31(6):1275–84. doi: 10.1111/1365-2435.12831 (PMC5518763; doi:10.1111/1365-2435.12831)

Contrasting effect of climate and population density over time and life-stages in a long-lived seabird.

Rémi Fay^1^, Christophe Barbraud ^1^, Karine Delord ^1^ and Henri Weimerskirch^1^

^1^ Centre d’Etudes Biologiques de Chizé, UMR 7372 CNRS/Univ La Rochelle, 79360 Villiers-en-Bois, France

Online supporting information

Appendix S1: Sex assignment

We used the life histories of 9685 individuals ringed as chicks and resighted between 1965 and 2013. Individuals were sexed in the field based on sexual size and plumage dimorphism, courting and mating behaviours. Since 1999 genetic assignments were available and were systematically used from 2003 (Weimerskirch, Lallemand & Martin 2005). Sex was known for 4913 birds (2431 females and 2482 males), including 2554 from observation (1264 females and 1290 males), and 2359 from genetic (1167 females and 1192 males). Of the remaining unsexed birds, 96.5 % were never seen after fledging on Possession Island, and can be considered as dead before recruitment (Charmantier et al. 2011). As all individuals needed to be included in the model in order to avoid overestimating survival, we inferred the sex of the unsexed birds using a binomial random distribution as in Pardo, Barbraud & Weimerskirch (2013). Knowing that our study population showed an equilibrium sex ratio at fledging (n=3126, p-value=0.99) and that the sex ratio of recaptured birds was also unbiased (n=3085, p-value=0.43), we deduced that the large majority of these birds never seen at the colony were individuals of both sexes in equal proportion. Thus, we were confident that this sex-inference procedure did not introduce spurious patterns since almost all individuals had the same life history (i.e. seen as chick and never recaptured) with a deductible sex-ratio of 1. To valid our process, we repeated the random sex assignment 10 times to check the stability of the sex specific survival parameters.

References

Charmantier, A., Buoro, M., Gimenez, O. & Weimerskirch, H. (2011) Heritability of short‐scale natal dispersal in a large‐scale foraging bird, the wandering albatross. *Journal of evolutionary biology*, 24, 1487-1496.

Pardo, D., Barbraud, C., & Weimerskirch, H. (2013) Females better face senescence in the wandering albatross. *Oecologia*, 173, 1283-1294.

Weimerskirch, H., Lallemand, J. & Martin, J. (2005) Population sex ratio variation in a monogamous long-lived bird, the wandering albatross. *Journal of Animal Ecology*, 74, 285-291.

Appendix S2: Parametrization of the general model and biological constraints

Several biological constraints were applied to improve parameter identifiability. The initial state was constrained to the pre-recruitment state because all birds were banded as chick. Then, from the first year of life to the fifth, transition probability to the pre-recruitment stage was fixed to 1 because no recruitment occurred before 6 year-old. To model the pre-recruitment period we defined two main stages: the *juvenile* stage, which was an unobservable state, corresponding to the first 2 years of life spent continuously at sea (i.e. no individual of 1 or 2 year-old were observed at the colony), and the *immature* stage corresponding to non-recruited birds older than 2 years that started to visit the colony and could be potentially observed. Based on previous results provided by Fay et al. (2015), the immature stage was decomposed in three age classes: 3-8 years, 8-13 years and >13 years. Juvenile survival was set to be cohort dependent and both juvenile and immature survival rates were assumed to be sex-dependent. Recapture probability of the immature stage was modelled as age dependent to fit the progressive return of individuals at the breeding colony before recruitment. From 6 years of age birds may recruit and move toward the mature states SB and FB depending both on sex and age. Recruitment rate was constrained to be constant after age 10 (Fay et al. 2015). For mature birds, survival was assumed to be sex-dependent (Barbraud and Weimerskirch 2012). Based on Pardo, Barbraud & Weimerskirch (2014), we distinguished different survival probabilities for breeders and post reproductive breeders on one side and recruited non-breeders on the other side. Note that we were not able to model actuarial senescence as in Prado et al. (2013) due to a very small sample size of old individuals with known aged parents. Transitions were set to be state dependent. Since wandering albatrosses are monogamous and both sexes exhibit a quasi-biennial breeding, transitions were constrained to be similar between sexes. Recapture probabilities were assumed to be state dependent due to lower detection probability for observable non-breeders and failed breeders compared to successful breeders. Finally, our general model was:

$$\Phi_{a_{(1to2)}.sex.cohort, a_{(3to8,9to13,13+)}.sex}^{pre}\Phi_{sex.state}^{ad}\Psi_{a.sex}^{pre\to ad}\Psi_{cst}^{ad\to ad} p_{a}^{pre}p_{state}^{ad}$$

where the pre-recruitment (pre) survival probability (Φ) was age-class (a), sex and cohort dependent, the adult (ad) survival probability was sex and state dependent, the probability of transition (Ψ) from pre-recruitment to adult was age and sex dependent, the probability of remaining in the adult stage was constant (cst), the pre-recruitment capture probability (p) was age dependent, and the adult capture probability was state dependent. In this model notation, symbols “.” indicate interactive effects, “1to2”, “3to8” and “9to13” indicate that age classes were grouped and “>13” indicates that age classes were grouped after 13 years.

References

Barbraud C. and Weimerskirch, H. 2012 Estimating survival and reproduction in a quasi-biennially breeding seabird with uncertain and unobservable states. *Journal of Ornithology.* 152, 605–615.

Fay R., Weimerskirch H., Delord K. and Barbraud, C. 2015 Population density and climate shape early-life survival and recruitment in a long-lived pelagic seabird. *Journal of Animal Ecology* 84, 1423–1433.

Pardo, D., Barbraud, C. and Weimerskirch, H. 2013 Females better face senescence in the wandering albatross. *Oecologia* 173, 1283–1294.

Pardo D., Barbraud C. and Weimerskirch, H. 2014 What shall I do now? State-dependent variations of life-history traits with aging in Wandering Albatrosses. *Ecology and Evolution* 4, 474–487.

Table S1 - Results from the GOF tests performed on the adult component of the dataset with known states.

|  | Females | | | Males | | |
| --- | --- | --- | --- | --- | --- | --- |
| Test | χ2 | df | P | χ2 | df | P |
| WBWA | 192.3 | 136 | 0.001 | 251.8 | 194 | 0.003 |
| 3G.SR | 156.5 | 98 | <0.001 | 244.9 | 100 | <0.001 |
| 3G.Sm | 571.7 | 471 | 0.001 | 688.6 | 547 | <0.001 |
| M.ITEC | 262.6 | 110 | <0.001 | 359.8 | 161 | <0.001 |
| M.LTEC | 82.9 | 44 | <0.001 | 78.4 | 57 | 0.032 |

GOF tests indicated that some assumptions of the JMV model were not supported. First, test M.ITEC indicated the presence of trap-dependence (Pradel et al. 2005). This was clearly due to the quasi-biennial breeding strategy of wandering albatrosses, which is known to create trap-shyness in GOF tests (individuals seen breeding in year t are less likely to be seen in year t+1 due to their sabbatical year). However, given that our general model structure included unobservable states to explicitly take into account quasi-biennial breeding, we ignored the M.ITEC component in the GOF test of the general model. Second, although we removed the first capture corresponding to the fledging stage, test 3G.SR indicated the presence of transience. This was partly due to young individuals visiting the colony for the first time and never seen again. Finally, test WBWA indicated that individuals tended to remain in the same state from one occasion to the other. We thus estimated the GOF of the general model by summing the components WBWA, 3G.SR, 3G.Sm and M.LTEC of the GOF tests and used a variance inflation factor. Note that since the general model included age effects on survival, our model selection is conservative since these age effects partly accounted for transience.

Figure S1 - Distribution area from which SSTA values were extracted for (a) juvenile (1-2years), (b) immature (>2years) and (c) parents distinguishing male range (solid square) from female range (dashed square).


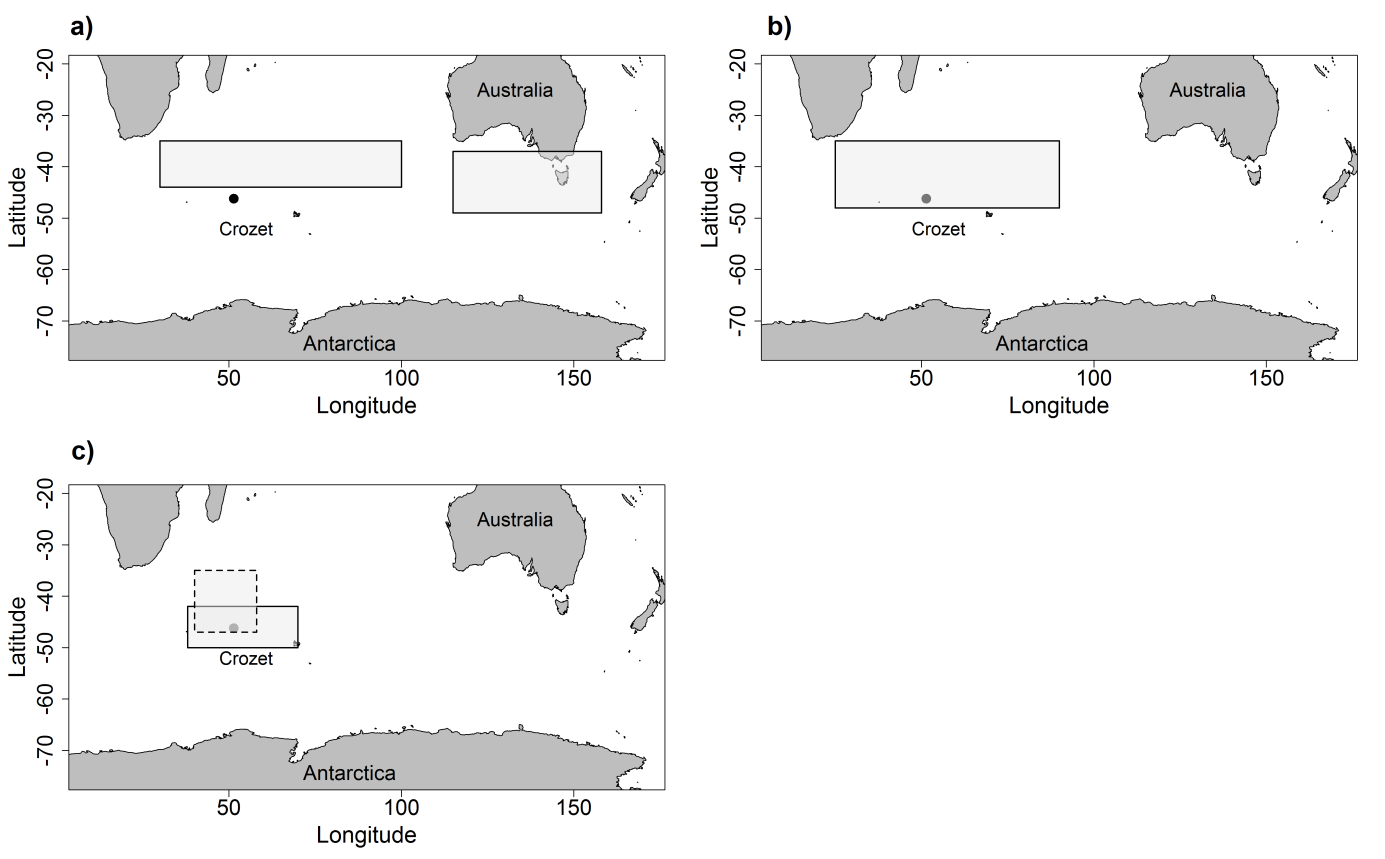


Figure S2 – Estimated cumulated probability to be recruited according to age and sex for the wandering albatross population of Crozet. Open and filled dots stand for males and females respectively. Estimates are calculated from age specific recruitment probability by bootstrapping methods (1000 simulations). Standard errors estimate are of the same magnitude that dot size.


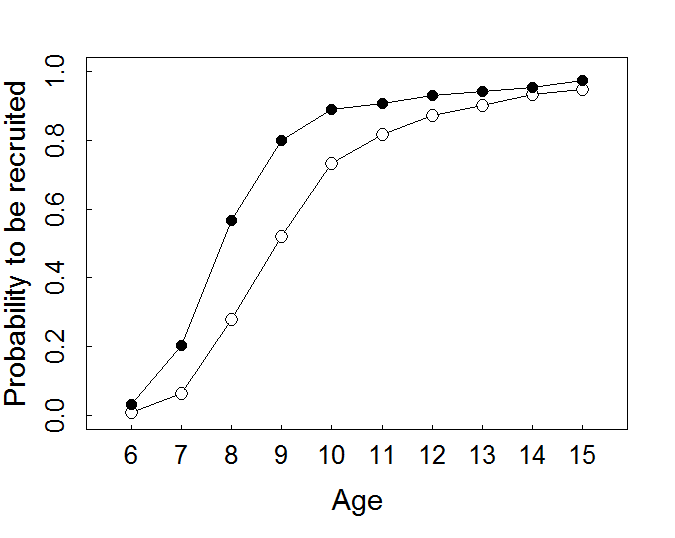

Supplement: Supplementary file 2 — Appendix S1. Sex assignment. Appendix S2. Parametrization of the general model and biological constraints. Fig. S1. Distribution area from which SSTA values were extracted. Fig. S2. Cumulated probability to be recruited. Table S1. GOF tests results. [file FEC-31-1275-s002.docx]
